# Fisher encoding of convolutional neural network features for endoscopic image classification

**DOI:** 10.1117/1.JMI.5.3.034504

**Published:** 2018-09-24

**Authors:** Georg Wimmer, Andreas Vécsei, Michael Häfner, Andreas Uhl

**Affiliations:** a University of Salzburg, Department of Computer Sciences, Salzburg, Austria; b St. Anna Children’s Hospital, Vienna, Austria; c St. Elisabeth Hospital, Vienna, Austria

**Keywords:** colonic polyps, celiac disease, convolutional neural networks, Fisher encoding, endoscopy

## Abstract

We propose an approach for the automated diagnosis of celiac disease (CD) and colonic polyps (CP) based on applying Fisher encoding to the activations of convolutional layers. In our experiments, three different convolutional neural network (CNN) architectures (AlexNet, VGG-f, and VGG-16) are applied to three endoscopic image databases (one CD database and two CP databases). For each network architecture, we perform experiments using a version of the net that is pretrained on the ImageNet database, as well as a version of the net that is trained on a specific endoscopic image database. The Fisher representations of convolutional layer activations are classified using support vector machines. Additionally, experiments are performed by concatenating the Fisher representations of several layers to combine the information of these layers. We will show that our proposed CNN-Fisher approach clearly outperforms other CNN- and non-CNN-based approaches and that our approach requires no training on the target dataset, which results in substantial time savings compared with other CNN-based approaches.

## 1 Introduction

Convolutional neural networks (CNNs) are gaining more and more interest in computer vision. CNNs widely replaced “shallow” (nondeep learning based) image representations, such as Fisher vectors, vector of locally aggregated descriptors (VLAD), or the bag-of-words (BoW) approach, which were previously state of the art.^1^ Also in case of the automated diagnosis of endoscopic images, CNNs outperformed shallow image representations.^2^^,^^3^

The most common ways to employ CNNs for image retrieval and image classification tasks are to employ the CNN inbuilt classifier (e.g., SoftMax classifier) or to use global image features extracted from fully connected (FC) CNN layers (FC layer activations) and classify them using support vector machines (SVMs).

In a simplified way, CNNs consist of filter banks stringed together with a classifier at the end. The activation of the first convolutional layer is nothing more than the convolution with a filter bank and also the activations of the following CNN layers are just filter responses of filter responses, which were potentially nonlinearized [rectified linear unit (ReLU)] and pooled. The layer activations of FC layers can be directly used for classification (e.g., SoftMax or SVM classifiers), but the activations of the convolutional layers are in general too high dimensional to be directly used for classification, and so feature extraction methods or dimensionality reduction methods need to be applied to those CNN activations prior to classification.

Recent research efforts have shown that combining CNNs with shallow image representations can outperform CNNs alone in various applications. The most common combination of CNNs and shallow image representations in the literature is to apply shallow image descriptors to the activations of the nets. Although most approaches apply shallow image representations to the global image features extracted from FC CNN layers (e.g., VLAD pooling^4^ and Fisher vector encoding^5^ applied to CNN FC layer activations of image patches), a newer and less common approach is to apply the shallow image representations to the activations of the convolutional layers that represent local image information.

For example, Fisher vector encoding was applied to the activations of the last convolutional layer of a CNN for event detection in videos^6^ and for histopathology image classification.^7^ Further examples are to apply BoW to the activations of the third last convolutional layer of a net^8^ or to sum up the CNN activations for each filter in a convolutional layer.^9^ The approach summing up CNN activations is the only approach we found in previous literature that applies image descriptors to the earlier layers of a CNN, which include more generic features (e.g., edge detectors or color blob detectors). All other approaches that apply shallow image representations to CNN activations use the more class-specific later CNN layers (FC layers or later convolutional layers).

The classification of celiac disease (CD) and colonic polyps (CP) can be considered as a texture classification problem and it has been shown that filter banks are well suited for this task (e.g., the MR8 filter bank^10^ or wavelets^11^). Filter banks are shown to be suited for the classification of CD and CP; also, the layer activations of the early convolutional layers (which are basically just the outputs of stringed together filter banks) could contain valuable (local) information that enables a distinction between different stages of CD or CP.

In this work, we propose an approach applying improved Fisher vectors (IFV^12^) to the activations of early CNN convolutional layers followed by linear SVM classification. CNNs are state of the art endoscopic image classifications. Also image encoding methods such as Fisher encoding or BoW applied to local feature descriptors have been shown to be well suited for the classification of endoscopic images [e.g., BoW^13^ or IFV^2^ computed from scale-invariant feature transform (SIFT) descriptors]. In this work, we combine CNNs and IFV by applying IFV to local CNN feature descriptors (CNN convolutional layer activations). We will show in this work that applying Fisher encoding to CNN activations is more suited for the classification of endoscopic images than using other image representations that were applied to CNN activations in the literature.

An advantage of using features from early CNN layers is that those features are less class specific than features from later CNN layers. In our proposed approach, we extract information of the more generic early convolutional layers and so chances are higher that good results are achieved with nets pretrained on huge databases without any additional training of the nets on our endoscopic image databases. This would save a lot of computation time needed to train the nets and would solve the problem that our endoscopic image databases are too small to properly train CNNs without overfitting to the training data.

In our experiments, three different network architectures are utilized and for each network architecture, we use one version of the net that is pretrained on the ImageNet database without any adaption to the endoscopic data as well as one version of the net that is further trained on a specific endoscopic image database.

This work presents two contributions:

• We propose an image descriptor applying Fisher vector encoding to CNN activations of earlier convolutional layers. There are no previous publications that apply Fisher encoding to the activations of the early and less class-specific convolutional layers. We will show that applying Fisher vector encoding to the activations of early convolutional layers achieves better results in general than applying it to later convolutional layers.
  Common CNN features such as CNN SoftMax classification and the SVM classification of FC layer activations are state of the art in endoscopic image classification and outperform shallow image representations as shown in the literature. We will show that our proposed image representation outperforms common CNN features, shallow image representations as well as other combinations of CNNs and shallow image representations on our three endoscopic image databases (one CD database and two CP databases). A further advantage of our approach is that it requires no training of the nets (contrary to common CNN features such as CNN SoftMax classification and the SVM classification of FC layer activations applied to endoscopic image classification^2^), which results in substantial time savings.
• We combine the information from multiple CNN convolutional layer activations by concatenating the Fisher representations of these layers. To the best of our knowledge, there are no previous publications applying image descriptors to convolutional layer activations that combine the information from multiple convolutional layer activations. We will see that this step distinctly improves the results.

## 2 Celiac Disease and Colonic Polyps

Endoscopy with biopsy is currently considered the gold standard for the diagnosis of CD and CP. Computer-assisted systems for the diagnosis of CD and CP have potential to improve the whole diagnostic workup by saving costs, time, and manpower and at the same time increasing the safety of the procedure.

### 2.1 Celiac Disease

CD is a multisystemic immune-mediated disease, which is associated with considerable morbidity and mortality.^14^ In untreated or inappropriately treated CD, the inflammation caused by the dysregulated immune response can disrupt the intestinal mucosa thus leading to a total atrophy of the villi (finger-like projections of the mucosa) which causes a diminished ability to absorb nutrients. More than two million people in the United States, this is about one in 133, have this disease.^15^

**Fig. 1:**
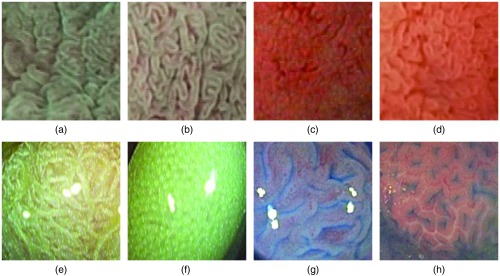
In the top row, we show example images of the CD database for the two classes healthy and CD using ${\mathrm{NBI}}_{\mathrm{MIT}}$ as well as ${\mathrm{WL}}_{\mathrm{MIT}}$ endoscopy. In the bottom row, we show example images of both classes of CP from the HD database (left side) and the HM database (right side). (a) ${\mathrm{NBI}}_{\mathrm{MIT}}$, CD, (b) ${\mathrm{NBI}}_{\mathrm{MIT}}$, healthy, (c) ${\mathrm{WL}}_{\mathrm{MIT}}$, CD, (d) ${\mathrm{WL}}_{\mathrm{MIT}}$, healthy, (e) HD neoplastic, (f) HD non-neoplastic, (g) HM neoplastic, and (h) HM non-neopl.

For the automated diagnosis of CD, we differentiate between healthy mucosa and mucosa affected by CD using an image database consisting of images gathered by the modified immersion technique (MIT^16^) using traditional white-light illumination (denoted as ${\mathrm{WL}}_{\mathrm{MIT}}$) as well as narrow band imaging (denoted as ${\mathrm{NBI}}_{\mathrm{MIT}}$). Examples of the two classes for both imaging modalities are shown in Figs. 1(a)–1(d). It was shown that using ${\mathrm{NBI}}_{\mathrm{MIT}}$ or ${\mathrm{WL}}_{\mathrm{MIT}}$ as imaging modality has a significant impact on the underlying feature distribution of general purpose image representations.^17^ However, it was also shown that systems trained on images from both modalities generalize well without requiring additional domain adaption techniques and that combining both modalities improves the accuracies in case of an insufficient amount of data for training (as is probably the case for CNNs).

Prior works dealing with the computer-aided diagnosis of CD employed general purpose image descriptors such as local binary pattern (LBP)-based operators^18^^,^^19^ as well as wavelet-transform based features.^20^^,^^21^ Furthermore, methods specifically designed for the diagnosis of CD were developed such as shape features^22^ describing the curvature in mucosal structure, bandpass-type Fourier filters^23^ extracting features from bandpass filtered images, and fractal analyze-based features.^10^ In general, contrast sensitive image descriptors turned out to be well suited for the automated diagnosis of CD because the most distinctive visual difference between healthy and abnormal images is the partly or entirely missing of the villi in case of areas affected by CD. Thus, images of affected areas have less contrast than images showing healthy mucosa [see Figs. 1(a)–1(d)]. Recently, CNNs were applied for the diagnosis of CD and outperformed previously used image descriptors.^2^^,^^24^ There is also an survey on computer-aided decision support for the diagnosis of CD.^25^

### 2.2 Colonic Polyps

CPs are a rather frequent finding and are known to either develop into cancer or to be precursors of colon cancer. Hence, an early assessment of the malignant potential of such polyps is important as this can lower the mortality rate drastically.

For the automated diagnosis of CP, we differentiate between two classes, normal mucosa or hyperplastic polyps (class non-neoplastic) and neoplastic, adenomatous, or carcinomatous structures (class neoplastic). Experiments are applied on two CP databases. One CP image database is gathered by high-definition (HD) endoscopy in combination with the i-Scan technology (further denoted as HD database) and the second CP image database is gathered by high-magnification (HM) endoscopy in combination with chromoscopy (further denoted as HM database). Example images of the two classes for both CP databases are shown in Figs. 1(e)–1(h).

In the following, we give an overview on prior works on the computer-assisted staging of CP using HD endoscopic image databases or HM endoscopic images databases:

1. HD endoscopy:
  Prior works employed general purpose image descriptors such as wavelet-based features^26^ and features especially developed for the classification of CP such as fractal analysis-based features,^26^ specifically developed filter banks,^27^ and shape and contrast features extracted from segmented blobs modeling the pit-pattern structure.^28^ Recently, it was shown that CNNs outperform handcrafted features.^3^
2. HM endoscopy:
  Prior works employed general purpose image descriptors such as LBP,^29^ wavelet-based features,^11^^,^^30^^,^^31^ and SIFT features in combination with BoW.^13^ Furthermore, methods specifically designed for the classification of CP were developed, estimating the pit density using Delaunay triangulation^32^ or describing the structure of segmented blood vessel structure on endoscopic images^33^ (which performed quite well but is only suited for images gathered by NBI). The pit-pattern structure of the polyps is the most important feature to distinguish between different types of polyps for most of the mentioned methods (except of the image descriptor analyzing the blood vessel feature).

## 3 Material and Methods

### 3.1 CNN Training and Feature Extraction

This section gives the implementation details for CNN training and CNN feature extraction as well as the description of the employed nets.

In this work, three different CNNs are employed, the AlexNet,^34^ the VGG-f net,^1^ and the VGG-16 network.^35^ AlexNet and the VGG-f net both consist of five convolutional layers and three FC layers with a final SoftMax classifier. The VGG-16 net consists of 13 convolutional layers subdivided in five convolutional blocks (where each of the two to three convolutional layers inside of a block has the same number and sizes of filters) and three FC layers with a final SoftMax classifier.

As already mentioned in Sec. 1, we apply two CNN learning strategies.

For the first learning strategy, the CNNs are trained on a specific endoscopic image database. As initialization for the convolutional layers of the AlexNet and VGG-f net, we use the parameters that were learned on the ImageNet data. In case of the VGG-16 net, the convolutional parameters are randomly initialized^36^ as the VGG-16 net did not work well using pretrained coefficients as initialization. As the FC layers are more specific to the details of the classes contained in the ILSVRC ImageNet challenge data, we randomly initialize the coefficients of those layers instead of using the parameters that were learned on the ImageNet data. The size of the last FC layer is adapted to our two-class classification scheme, which means that the size of the convolutional filters is changed from 1×1×4096×1000 for the ImageNet to 1×1×4096×2. The last FC layer is acting as SoftMax classifier and computes the training loss (log-loss). Training is performed on batches of 128 images each, which are for each iteration randomly chosen from the training data and subsequently augmented (see Sec. 3.4). Stochastic gradient descent with weight decay (λ=0.0005) and momentum (μ=0.9) is used for the training of the models.

For the second learning strategy, we use the CNNs that were pretrained on the ImageNet ILSVRC challenge data as fixed feature extractors without any training on our CD or CP datasets.

For both learning strategies, the activations of CNN convolutional layers are extracted as features for further Fisher encoding. That means the images are fed through the networks and the outputs of convolutional layers [without pooling or nonlinearization (ReLU)] are used for Fisher encoding.

### 3.2 Fisher Encoding of CNN Activations

This section gives the implementation details for applying IFV encoding on the activations of CNN convolutional layers.

**Fig. 2:**
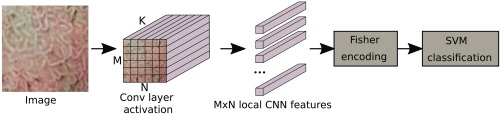
Scheme of our proposed CNN-Fisher approach.

IFVs^12^ are based on estimated Gaussian mixtures of local image descriptors. The improved version based on a nonlinear Hellinger’s kernel and $l^{2}$ normalization with 16 clusters is used. Each activation of a convolutional layers consists of K different M×N (M=N in our case) feature maps, where K is the number of filters kernels in the convolutional layer and M×N is the size of the filter response for each filter kernel. These feature maps can be viewed as N×M local descriptors of dimension K. The IFV image representation is obtained by pooling those M×N local descriptors for a given activation of a convolutional layer. The IFV representation of an convolutional layer is a vector of length K×2×16 [16 Gaussian mixture model (GMM) clusters and two parameters (mean and covariance)]. Figure 2 shows the pipeline of our CNN-Fisher approach from the CNN feature acquisition to the Fisher representation and to the final SVM classification.

The reason why we use 16 GMM clusters for Fisher encoding is that the number of local CNN features is already quite low for the last convolutional layer [169 (M=N=13, M*N=169) for VGG-f and AlexNet and 196 (M=N=14, M×N=196) for VGG-16] and hence a too high number of clusters would not make sense. We want to use the same number of clusters for each convolutional layer and so using 16 clusters is an appropriate choice. We also perform experiments with 8 and 32 clusters, but, if not mentioned otherwise, 16 clusters are used.

We do not only classify the extracted IFV features of single convolutional layers, we also combine the information of several different convolutional layers by concatenating the IFV feature vectors of those layers. More specifically, in case of the AlexNet and VGG-f net we separately classify the IFV representation of each layer yielding in one classification result per layer. Additionally, we concatenate the IFV representations from the first i convolutional layers with i∈{2,…,5} and further classify the concatenated feature vectors [and so we get one result for the combination of the first two convolutional layers (i=2), one for the first three (i=3), one for the first four (i=4), and one for the combination of all convolutional layers (i=5)].

In case of the VGG-16 net, we proceed analogously and separately classify the Fisher representations of the first convolutional layer of each convolutional block (the VGG-16 net consists of five convolutional blocks with two to three convolutional layers per blocks) and additionally we concatenate the Fisher vector representations of the first convolutional layers from the first till the i’th convolutional block with i∈{2,…,5} for further classification.

So, for example, the IFV representation of the second convolutional layer of the VGG-16 net is 4096 dimensional [128×2×16 (K=128, 2 parameters and 16 GMM clusters)] and the concatenation of the IFV representations from all five layers is 47,104 dimensional [(64+128+256+512+512)×2×16].

### 3.3 Comparison Methods

To compare the results of our proposed CNN-Fisher approach with results of other approaches, we additionally perform experiments using five CNN-based approaches and four handcrafted image descriptors. The nine methods are either state of the art in the automated diagnosis of CD or CP or they are related to the CNN-Fisher approach. For the five CNN-based approaches, we apply the same nets as for our proposed approach to enable a direct comparison with the CNN-Fisher approach. All comparison methods except of one (CNN SoftMax Classification) are classified using linear SVMs.

#### 3.3.1 CNN SoftMax classification and CNN SVM classification

The classification of images using the CNN built-in SoftMax classifier and by applying SVMs to the FC layer activations are the two most common CNN classification approaches and are state of the art in the automated diagnosis of CD^2^ and CP.^3^

#### 3.3.2 Bag of words

The BoW approach applies BoW encoding to the activations of the last convolutional layer. So similar to our proposed approach, this approach applies a shallow image descriptor to CNN convolutional layer activations.^8^

#### 3.3.3 Vector of locally aggregated descriptors

Also, this approach applies a shallow image descriptor to CNN activations. The VLAD approach performs VLAD pooling to FC layer activations from different patches of an image.^4^

As proposed in the literature, the two methods such as BoW and VLAD are only applied to pretrained nets (trained on the ImageNet database) and not to the nets adapted to the endoscopic databases.

#### 3.3.4 Sum

The sum approach sums up the intensities of the CNN activations from the third convolutional layer. More specifically, the feature maps of each filter kernel of the third convolutional layer are summed up and those sums form the feature vector used for classification. This approach was designed to detect and classify CP.^9^

#### 3.3.5 Dual-tree complex wavelet transform

The dual-tree complex wavelet transform (DT-CWT)^11^ is a multiscale and multiorientation wavelet transform. The DT-CWT is applied using four decomposition levels. The feature extraction step is based on fitting a two-parameters Weibull distribution to the coefficient magnitudes of the DT-CWT sub-bands. It was shown that this method is highly suited for the classification of CP based on HD as well as HM endoscopic images.^11^

#### 3.3.6 Blob shape adapted gradient local fractal dimension

The blob shape adapted gradient local fractal dimension (BSAGLFD) approach combines features that describe the shape of the pit-pattern structure, a contrast feature, and a fractal analysis-based feature that analyzes changes in the intensity distribution in expanding regions. It was shown that this method is highly suited for the classification of CP based on HD as well as HM endoscopic images.^26^

#### 3.3.7 Fisher SIFT

The Fisher SIFT approach^12^ applies IFV encoding to SIFT descriptors computed on a dense 6×6 pixel grid. So this approach is similar to our proposed approach, but IFV is applied to local SIFT features instead of local CNN features. This approach has already proven to be suited for the classification of CD;^2^ however, its results were worse than those of CNNs.

#### 3.3.8 Bag-of-words SIFT

The BoW SIFT approach applies BoW to SIFT descriptors computed on a dense 5×5 pixel grid. This approach has proven to be suited for the classification of CP based on HM endoscopic imagery gathered using NBI.^13^

### 3.4 Experimental Setup

The CD database consists of 1661 RGB image patches of size 128×128 pixels that are gathered by means of flexible endoscopes using ${\mathrm{NBI}}_{\mathrm{MIT}}$ as well as ${\mathrm{WL}}_{\mathrm{MIT}}$. About 1045 images are gathered by ${\mathrm{WL}}_{\mathrm{MIT}}$ endoscopy (587 healthy images and 458 affected by CD) and 616 images are gathered by ${\mathrm{NBI}}_{\mathrm{MIT}}$ endoscopy (399 healthy images and 217 affected by CD). So, in total 986 image patches show healthy mucosa and the remaining 675 image patches show mucosa affected by CD. The images were captured from 353 patients. As our used nets require input image sizes of 224×224×3 (VGG-f net and VGG-16 net) respectively 227×227×3 (AlexNet), the images of the CD database are bicubicly upscaled to a size of 256×256×3 to be able to extract patches of the images for training and classification.

The HD database was acquired by extracting patches of size 256×256×3 from frames of HD-endoscopic (Pentax HiLINE HD+ 90i Colonoscope) videos. Our database consists of patches gathered with four different imaging modalities [three different i-Scan modes (modes 1, 2, and 3) and no i-Scan]. The HD database consists of 478 image patches (144 images showing non-neoplastic mucosa and 301 images showing neoplastic polyps) from 84 patients.

The HM database is acquired using a zoom-colonoscope (Olympus Evis Exera CF-Q160ZI/L) with a magnification factor of 150 and indigocarmine dye-spraying. The database is acquired by extracting 716 patches (198 non-neoplastic and 518 neoplastic) of size 256×256×3 from 327 endoscopic color images of 40 patients.

The images of all three databases are extracted only from regions for which histological findings are available.

The image data is normalized by subtracting the mean image of the training portion for each database. We then linearly scale each image within [−1,1].

Due to the small amount of available data, we use data augmentation to increase the number of images for the training of the nets. For each training iteration, a batch of images is randomly chosen from the training data. The augmentation is based on cropping a subimage [227×227 (AlexNet), respectively, 224×224 pixels (VGG-f and VGG-16)] from each image of a batch with randomly chosen position. Subsequently, the subimage is randomly rotated (0 deg, 90 deg, 180 deg, or 270 deg) and randomly either flipped or not flipped around the horizontal axis. Training is performed for 5000 (AlexNet and VGG-f net) and 10,000 (VGG-16 net) iterations, respectively. Validation is performed using the central patch of an image and no data augmentation techniques are applied to the validation images.

Because of the relatively small amount of data, we perform fivefold cross validation to achieve a stable estimation of the generalization error. For each of the fivefolds, we took care that images of a single patient are never in training and evaluation sets. All nets and comparison methods are trained using the training portion of our data corpus and we use the same folds for each method. The final validation is performed on the left-out part. That means for each network architecture and each image database, we train five different nets, one for each of the fivefolds (except for the case where we use the CNNs as fixed feature extractor, where no training is performed). In our experiments, we compute the overall classification rate (OCR) for each fold and report the mean OCR over all fivefolds. For the comparison of the results from our CNN-Fisher approach with the results of other state-of-the-art approaches, we additionally report the standard deviation and the range of OCRs over the fivefolds.

For each of our databases, we use the same training and evaluation set portions for all methods.

The CNNs are implemented using the MatConvNet framework^37^ and the SVMs are provided by the LIBLINEAR library.^38^ We use a linear one-class SVM and the SVM cost factor (C) is found using cross validation on the training data. SVM classification is applied to our proposed method as well as to all comparison methods except for the CNN SoftMax classification. We use the implementation of IFV as provided by VLFeat.

## 4 Results and Discussion

### 4.1 Results of the Convolutional Neural Network-Based Methods

The results of the experiments using our three nets trained on the endoscopic image databases, respectively, on the ImageNet database (IN) are shown in Table 1. Our proposed CNN-Fisher approach is applied using either the Fisher representations of single (CNN-Fisher SL) and multiple convolutional layers (CNN-Fisher ML), whereat $C_{i}$ denotes the i’th convolutional layer and $C_{1,\ldots ,i}$ denotes the combination of the first i convolutional layers. As comparison methods, we employ the five CNN-based methods SoftMax classification (SM), the SVM classification of FC layer activations (FC), and the BoW, VLAD, and sum approach. We only show the results of the comparison approaches with the same training approach [training on the endoscopic image database or using a pretrained net (IN)] as used in the literature. The results for the other (not computed) training approaches are marked with a “−”. For each CNN, the best result over the different classification approaches is given in bold face numbers. To provide an overview of which CNN-based methods work best in general for endoscopic image classification, we additionally present the averaged results over the results of all databases and nets per method (Ø in Table 1). Once again we differentiate between nets trained on endoscopic image databases (E-IMDB) and nets trained on the ImageNet database (IN).

**Table 1:** Mean classification rates over the fivefolds. The CNNs are trained on a specific endoscopic database (the CD, HD, or HM database) or on the ImageNet database (IN). In the two bottom rows (Ø), we show the method’s average results over all databases and net architectures [three (databases) × three (net architectures) = nine results per method] for training on the endoscopic image databases (E-IMDB) as well as for training on the ImageNet database (IN).

	CNNs	Classification approaches													
		CNN comparison methods					CNN-Fisher SL					CNN-Fisher ML			
		SM	FC	BoW	Vlad	Sum	$C_{1}$	$C_{2}$	$C_{3}$	$C_{4}$	$C_{5}$	$C_{1,2}$	$C_{1,2,3}$	$C_{1,\ldots ,4}$	$C_{1,\ldots ,5}$
CD	AlexNet (CD)	85.5	86.2	—	—	—	81.7	83.0	85.2	86.1	85.4	86.5	87.9	88.2	**88.3**
	AlexNet (IN)	—	80.7	79.1	78.3	81.5	88.0	85.6	86.3	84.6	83.7	89.1	89.2	**89.3**	89.1
	VGG-f (CD)	89.6	89.9	—	—	—	89.5	87.8	88.7	89.2	88.6	91.2	**91.7**	91.6	91.5
	VGG-f (IN)	—	78.6	78.9	82.4	86.1	90.3	88.8	88.0	87.0	84.9	91.7	91.7	**91.9**	91.8
	VGG-16 (CD)	87.8	87.2	—	—	—	77.8	81.2	85.9	85.6	86.4	82.6	86.1	87.3	**87.9**
	VGG-16 (IN)	—	88.9	84.3	81.8	87.8	81.8	90.5	90.0	90.4	89.2	89.9	91.6	92.2	**92.5**
HD	AlexNet (HD)	82.9	82.3	—	—	—	**86.6**	80.2	81.3	82.2	83.0	86.4	85.2	85.3	85.2
	AlexNet (IN)	—	78.5	79.6	76.4	75.5	87.9	86.3	85.4	79.6	79.5	**88.0**	**88.0**	87.4	87.6
	VGG-f (HD)	86.7	86.0	—	—	—	89.6	87.9	88.1	88.5	87.3	89.4	**90.5**	90.0	89.8
	VGG-f (IN)	—	80.7	78.9	81.5	84.8	89.4	88.8	86.2	83.0	82.4	**90.7**	89.7	89.6	88.6
	VGG-16 (HD)	81.3	81.7	—	—	—	82.1	81.5	81.4	81.4	81.2	83.2	82.0	84.3	**84.8**
	VGG-16 (IN)	—	86.0	85.6	70.9	87.0	84.1	89.3	89.1	89.4	88.3	89.5	**91.2**	90.3	90.1
HM	AlexNet(HM)	68.8	71.8	—	—	—	79.6	73.3	74.0	71.8	68.9	**82.0**	81.4	78.9	76.5
	AlexNet (IN)	—	75.0	80.6	73.9	76.1	84.6	81.7	87.6	85.6	81.6	85.0	86.9	86.9	**87.7**
	VGG-f (HM)	80.1	80.3	—	—	—	91.3	86.4	83.6	80.6	80.8	**91.6**	89.8	86.5	84.9
	VGG-f (IN)	—	73.6	80.6	69.7	85.8	91.4	88.0	85.5	84.4	81.6	**93.7**	93.4	92.9	92.9
	VGG-16 (HM)	76.9	76.9	—	—	—	77.3	70.8	72.6	76.3	75.1	76.1	76.3	77.9	**77.3**
	VGG-16 (IN)	—	89.1	84.8	74.7	83.8	82.2	90.3	90.4	90.2	88.9	90.2	91.7	92.4	**92.8**
Ø	E-IMDB	82.2	82.5	—	—	—	83.9	81.3	82.3	82.4	81.9	85.4	**85.7**	85.6	85.1
	IN	—	81.2	81.4	76.6	83.2	86.6	87.7	87.6	86.0	84.5	89.8	**90.4**	90.3	90.3

As we can see in Table 1, the clearly best results for each net architecture and each database are achieved using our proposed CNN-Fisher approach using multiple convolutional layers and no adaption of the nets to the endoscopic databases. The overall best result for the CD database (92.5%) is achieved using the VGG-16 net trained on the ImageNet database using all convolutional layers ($C_{1,\ldots ,5}$), for the HD database the best result (91.2%) is achieved using the VGG-16 net trained on the ImageNet database with layers $C_{1,\ldots ,3}$, and for the HM database the best result (93.7%) is achieved using the VGG-f net trained on the ImageNet database with layers $C_{1,2}$.

So, combining the information of multiple convolutional layers distinctly improves the results compared with just using single layers. The later a CNN convolutional layer is in the net, the bigger is the receptive field size (the region in the input space that a particular CNN’s feature is looking at) of its extracted features. So, the combination of several layers leads to a multiscale representation of an image that clearly outperforms the single-scale representation using only one convolutional layer.

The best results for endoscopic image classification in general are achieved by our CNN-Fisher approach combining the information from three to five convolutional layers ($C_{1,2,3}$, $C_{1,\ldots ,4}$, and $C_{1,\ldots ,5}$). As we can see in Table 1 [Ø (IN)], these three configurations of the CNN-Fisher approach achieve average classification rates across all databases and nets of over 90% for nets trained on the ImageNet database. That means the number of incorrectly classified images is nearly halved in comparison with the best performing CNN comparison method (sum: 83.2%).

When we consider the CNN-Fisher results using single convolutional layers (CNN-Fisher SL), we can observe that the best results in general are achieved for the first three convolutional layers ($C_{1}$, $C_{2}$, and $C_{3}$). These three configurations of the CNN-Fisher approach achieve average classification rates across all databases and net architectures of about 87% for nets trained on the ImageNet database. So for the purpose of endoscopic image classification, the information contained in the earlier layer activations seems to be more suited than the information in the later convolutional layer activations. Applying Fisher encoding to the last convolutional layer activation $C_{5}$ (as proposed in Refs. 6 and 7) only achieves an average classification rate of 84.5% and the four CNN comparison methods only achieve average classification rates over all nets and databases of up to 83.2%.

When we take a look at the results of the FC activations, we can observe that training the nets on an endoscopic database generally improves the results compared with using the nets trained on the ImageNet database (like already showed in a previous work about the classification of CD^2^). Only the VGG-16 net performs better without training on an endoscopic database. We think that the VGG-16 net is too deep (too much parameters to learn) to be properly trained on our relatively small endoscopic images databases. The results of the SoftMax classifier are for all databases similar to those of the SVM classification of FC activations.

The results of the comparison methods BoW, VLAD, and sum are clearly worse than the results of our proposed approach. So, these three approaches combining shallow image representations with CNN activations are not as well suited for endoscopic image classification as our proposed approach. The VLAD approach is pooling the FC layer activations from patches of an image, but the endoscopic images mostly show homogeneous texture structures and so the patches of an image look quite similar. So this approach does make sense for object recognition (for which it was originally proposed), where objects stand out from the background, but apparently not for endoscopic images with their homogeneous texture structure. The BoW approach is quite similar to our proposed approach using the last convolutional layer $C_{5}$. The BoW approach applies BoW pooling and CNN-Fisher SL ($C_{5}$) applies Fisher pooling to the activations of the last convolutional layer. So, the only big difference between these two approaches is the pooling method (BoW versus Fisher). The results of CNN-Fisher SL ($C_{5}$) are about 3% higher in average than those of the BoW approach, which means that Fisher encoding is more suited than BoW encoding for our purpose. The sum approach is applied to the third convolutional layer ($C_{3}$). By comparing the results of the CNN-Sum approach with those of CNN-Fisher SL ($C_{3}$), we can see that that the results of CNN-Fisher SL ($C_{3}$) are >4% higher in average than those of the sum approach. So, Fisher encoding is more suited for the classification of endoscopic images than the more simple sum approach.

The highest differences in the results of the CNN-Fisher approach between the pretrained and the adapted version of a net architecture occur in case of the VGG-16 net, which was the only net architecture that was fully randomly initialized (contrary to the other two nets, where only the FC layers were randomly initialized and the parameters pretrained on ImageNet where used as initialization for the convolutional layers).

### 4.2 Comparison with State-of-the-Art Approaches

**Fig. 3:**
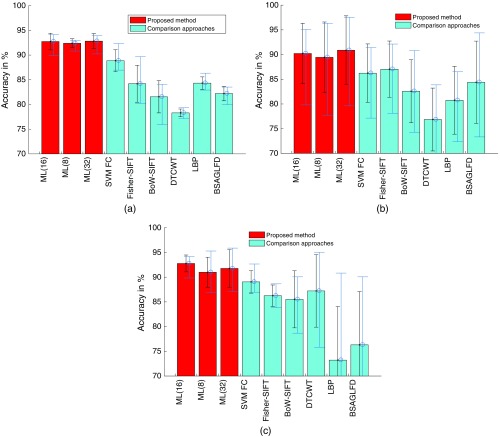
Results of our proposed CNN-Fisher approach with 16 (standard), 8, or 32 clusters [ML(16), ML(8), and ML(32)] compared with the results of five state-of-the-art approaches on our three endoscopic image databases. The bars show the mean classification rates over the fivefolds, the black error bars the standard deviations, and the blue error bars show the range of classification rates over the fivefolds. (a) CD, (b) HD, and (c) HM.

In Fig. 3, we compare the results of our proposed CNN-Fisher method using the best performing configurations [multiple layers ($C_{1,\ldots ,5}$) and the VGG-16 net trained on the ImageNet database] compared to five state-of-the-art approaches in the automated diagnosis of CD or CP (four shallow image descriptors and the SVM classification of CNN FC layer activations using the VGG-16 net). Our CNN-Fisher approach is additionally applied using 8 and 32 clusters for Fisher encoding (16 is the default parameter) to find out the optimal number of clusters.

As we can see in Fig. 3, our proposed method clearly outperforms other state-of-the-art methods on all three databases. We can observe that using 16 clusters for Fisher encoding achieves higher results than using only eight clusters. Using a higher number of clusters (32) does only slightly change the results and does not improve them overall.

We can observe in Figs. 3(b) and 3(c) that the classification rates are quite differing across the fivefolds for the two polyp image databases (note the big error bars). This is caused by the low number of patients in these two databases (HD:84 and HM:40). As all images of a patient have to be in the same fold, each fold contains only a very small number of patients. This, together with the fact that the images of some patients are easier/harder to classify than those of other patients, leads to the situation that some folds are easier/harder to classify than others. That is the main reason for the method’s high standard deviations and ranges of values over the fivefolds in case of the two polyp databases.

### 4.3 Comparison of the Training Approaches

**Fig. 4:**
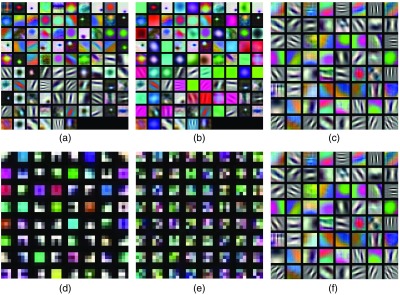
Convolutional kernels of the first convolutional layer learned on the ImageNet database or learned on our CD database (CDB). (a) AlexNet ImageNet, (b) AlexNet CDB, (c) VGG-f ImageNet, (d) VGG-16 ImageNet, (e) VGG-16 CDB, and (f) VGG-f CDB.

In Fig. 4, we see the convolutional kernels of the first convolutional layer of the three nets learned on the ImageNet as well as their convolutional kernels after training (the already pretrained nets) on our CD database, which is our image database with clearly the most images (1661).

In case of the VGG-16 net, which was fully randomly initialized, the filter kernels are entirely different for the CD version and the ImageNet version of the net. We can observe in Figs. 4(d) and 4(e) that the CD database is too small to train well-structured and smooth CNN filters and that is probably the reason why our proposed CNN-Fisher approach achieved distinctly higher results using the nonadapted ImageNet version of the VGG-16 net (see Table 1).

In case of the AlexNet and VGG-f net, we can observe that the training on the CD database only slightly changed the filter kernels. Only in case of the AlexNet, some of the filter kernels changed completely. Some of the filters of the AlexNet changed their colors [e.g., the filter on the right side of the third row in Figs. 4(a) and 4(b)] some other lose their entire structure and turned into simple averaging filters [e.g., the left filter in the first row in Figs. 4(a) and 4(b)]. We think that this loosing of structure could possibly be the reason for the higher results of our CNN-Fisher approach (for $C_{1}$) using the ImageNet version of the AlexNet. In case of the VGG-f net, the filter kernels undergo only very minor changes and also the differences between the CNN-Fisher results (for $C_{1}$) are only rather small between the adapted and the nonadapted version of the net. All the observations about changes of the filter kernels by training the nets on the CD database (based on Fig. 4) do also apply for the two other endoscopic databases (HD and HM).

So altogether that means our proposed method achieves higher results without training the nets on the endoscopic databases, whereas the widely used approach classifying FC layer activations generally performs better with nets trained on the endoscopic databases. The main reason for this behavior is that our CNN-Fisher approach uses the more generic information from the convolutional layers, whereas the approach classifying FC layer activations uses class-specific information from the FC layers (which requires training to the specific classes of the endoscopic database). In case of the VGG-16 net, an additional reason for the higher results using the nonadapted net is that the net is too deep to be properly trained on our small databases that results in overfitting to the training data and filter kernels that are neither smooth nor well structured.

So, a big advantage of our CNN-Fisher approach compared with the SVM classification of FC activations and the SoftMax classification is that we can save time by omitting the training of the nets.

### 4.4 Statistical Significance

**Fig. 5:**
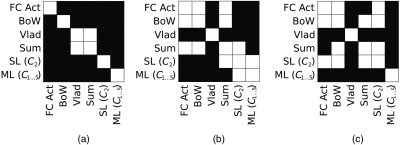
Result of the McNemar test for our three endoscopic image databases. A black square in a plot means that the two considered methods are significantly different with significance level of α=0.05. A white square means that there is no significant difference between the methods. (a) CD, (b) HD, and (c) HM.

By means of the McNemar test,^39^ we assess the statistical significance of our results from the CNN-based methods. With the McNemar test, we analyze if the images from a database are classified differently by the employed methods or if most of the images are classified identical. The McNemar test examines if the classification results of two methods are significantly different for a given level of significance (we use α=0.05) by building test statistics from incorrectly classified images. For the McNemar test, we employ the CNN-based comparison methods and both versions of our proposed approach (single layer and multiple layers) using the best performing combination of layers. That means we employ the second layer for the Fisher representation of single layers [SL ($C_{2}$) in Fig. 5] and all five layers for the Fisher representation of multiple layers [ML ($C_{1,\ldots ,5}$) in Fig. 5]. The McNemar test is applied for the methods using the VGG-16 net trained on the ImageNet database as this net achieved the best results over all three databases. The results of the MCNemar test on our three image databases are shown in Fig. 5. As we can see in Fig. 5, the CNN-based methods are generally significantly different. In case of the CD database, the CNN-Fisher approaches are significantly different than the other CNN-based methods. For the HD database, the CNN-Fisher approaches are significantly different than the other approaches except to each other and the sum approach [SL ($C_{2}$) compared to sum]. In case of the HM database, the multiple layer Fisher representation is significantly different than the other approaches, whereas the single-layer representation is only significantly different than the VLAD approach and the multiple layer representation.

## 5 Conclusion

In this work, we showed that our proposed CNN-Fisher approach is highly suited for endoscopic image classification. We showed that applying Fisher encoding to the earlier convolutional layer activations (that contain rather generic information) achieves higher results in general than applying Fisher encoding to the later convolutional layer activations, which contain more class-specific information. Combining the information of the Fisher representations of multiple convolutional layer activations further increased the results and clearly outperformed common CNN classification approaches and shallow image representations that are state of the art in endoscopic image classification. Also, the three comparison methods combining shallow image representations with CNN activations and the four shallow image representations were clearly outperformed by our proposed method. We showed that applying Fisher encoding to CNN convolutional layer activations is more suited than applying other image descriptors to the CNN activations (BoW and sum).

The best results were achieved using the combination of Fisher representations from different convolutional layers, which led to classification results up to 92.5% for the CD database, 91.2% for the HD database, and 93.7% for the HM database. It is interesting to note that the CNNs without any adaption to our endoscopic databases achieved higher results using our proposed CNN-Fisher approach than the CNNs trained on the endoscopic databases, whereas the quite common CNN classification technique using FC layer activations performs better if the nets are adapted to the endoscopic databases. So in addition to the better results, another advantage of the CNN-Fisher approach compared with standard CNN classification techniques (FC and SoftMax) is that the nets do not have to be trained anymore, which results in substantial time savings.
